# Acquired Asymptomatic Blue Tongue: A Report of Exogenous Agent-associated Tongue Dyschromia and Review of Blue Tongue Etiologies

**DOI:** 10.7759/cureus.6243

**Published:** 2019-11-27

**Authors:** Philip R Cohen

**Affiliations:** 1 Dermatology, San Diego Family Dermatology, San Diego, USA

**Keywords:** acquired, blue, congenital, dye, drug, dyschromia, gelato, lingual, medication, acquired, tongue, acquired, blue, congenital

## Abstract

This study presents the case of a man who developed a temporary and asymptomatic blue tongue. The dyschromia occurred following topical contact with gelato that contained Food, Drug, and Cosmetic (FD&C) blue dye no. 1. The etiology of a blue tongue is either congenital (in individuals with blue rubber bleb nevus syndrome) or acquired. Acquired blue dyschromia of the tongue results from either endogenous conditions or exogenous agents. The endogenous conditions include not only benign (angioleiomyoma, hemangioma, melanocytic macule, and varicosities) and malignant (ovarian carcinoma) tumors but also reactive lesions (intravascular papillary endothelial hyperplasia and mucocele) and systemic disorders (argyria, cyanosis, methemoglobinemia, primary adrenal insufficiency, and thrombocytosis). Exposure to the exogenous agents can either be systemic (ingestion of medications such as haloperidol, metoclopramide, minocycline, prochlorperazine, and risperidone), traumatic (tattoo resulting from the implantation of dental amalgam), or topical (contact with FD&C blue dye no. 1). Clinical clues to the topical exogenous etiology in the reported individual included not only the fact that the dyschromia spared both the lateral aspects and the tip of the tongue but also the observation that the blue color focally appeared on his upper lip.

## Introduction

Lingual dyschromia can be congenital or acquired. It can result from exogenous agents (received systemically, applied topically, or deposited traumatically), inherited syndromes, systemic conditions, benign melanocytic or vascular lesions, and reactive conditions. It is also rarely associated with visceral tumors [1-17]. This study describes the case of a man with acquired asymptomatic blue tongue from topical contact with Food, Drug, and Cosmetic (FD&C) blue dye no. 1 and reviews the potential etiologies of blue dyschromia of the tongue.

## Case presentation

A 60-year-old man presented with the new onset of an asymptomatic blue tongue. His friends had noted the change in the color of his tongue. He had been eating Cookie Monster simple gelato (Frozen Bean Corporation, Rancho Cucamonga, CA). The dry mix powder consists of not only FD&C blue no. 1 dye, but also several other ingredients: sugar, non-dairy creamer [non-hydrogenated coconut oil, corn syrup solids, sodium caseinate (a milk derivative), sugar, dipotassium phosphate, silicon dioxide, propylene glycol esters of fatty acids, monoglycerides and diglycerides, salt, soy lecithin, carrageenan, artificial flavor and annatto], chocolate cookies based cake {enriched flour [wheat flour, niacin, reduced iron, thiamine mononitrate (vitamin B1), riboflavin (vitamin B2), and folic acid], sugar, palm oil, and cocoa [processed with alkali]}, crystalline fructose, nonfat milk, sweet whey, cocoa powder (processed with alkali), guar gum salt, natural and artificial flavors, silicon dioxide (anti-caking agent).

**Figure 1:**
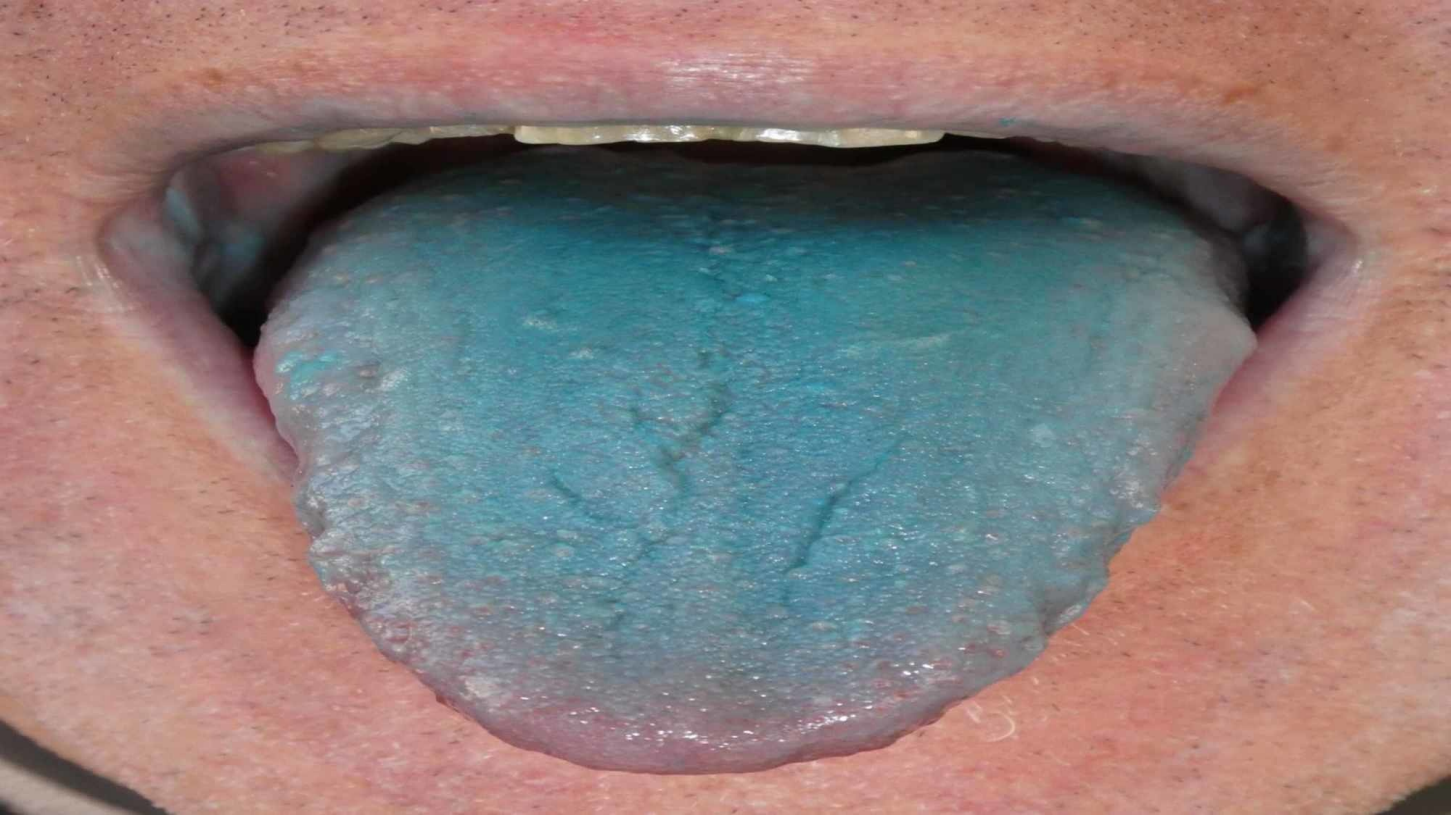
Food, Drug, and Cosmetic (FD&C) blue no. 1 dye-associated acquired asymptomatic blue tongue

An examination of his tongue showed that the dorsal surface had a confluent blue color (Figure 1). The distal tip and the lateral surfaces of the tongue retained their normal color. In addition, there was subtle blue discoloration of the mucosal upper lip.

The blue appearance of his tongue and upper lip spontaneously resolved within six hours. The dyschromia did not recur. Subsequent investigation determined that the composition of the gelato included FD&C blue dye no. 1.

## Discussion

A blue tongue is very uncommon. It is usually associated with an acquired condition. Endogenous and exogenous etiologies for blue lingual dyschromia are described below (Table 1) [1-17].

**Table 1:** Etiologies of a blue tongue CR: current report; FD&C: Food, Drug, and Cosmetic

Condition	Reference
Congenital conditions	[1]
Blue rubber bleb nevus syndrome	[1]
Acquired conditions	[2-17, CR]
Endogenous	[2-11]
Benign	[2-5]
Melanocytic macules	[2]
Vascular	[3-5]
Angioleiomyoma	[3]
Hemangioma	[4]
Varicosities	[5]
Malignant	[6]
Ovarian carcinoma	[6]
Reactive	[7,8]
Intravascular papillary endothelial hyperplasia	[7]
Mucocele	[8]
Systemic conditions	[6,9-11]
Argyria	[9]
Cyanosis	[10]
Methemoglobinemia	[11]
Primary adrenal insufficiency	[12,13]
Thrombocytosis	[6]
Exogenous	[14-17, CR]
Metal deposition	[14]
Amalgam	[14]
Systemic medication	[15-17]
Haloperidol	[15]
Metoclopramide	[15,16]
Minocycline	[17]
Prochlorperazine	[16]
Risperidone	[15]
Topical agent	[CR]
FD&C blue dye no. 1	[CR]

**Figure 2:**
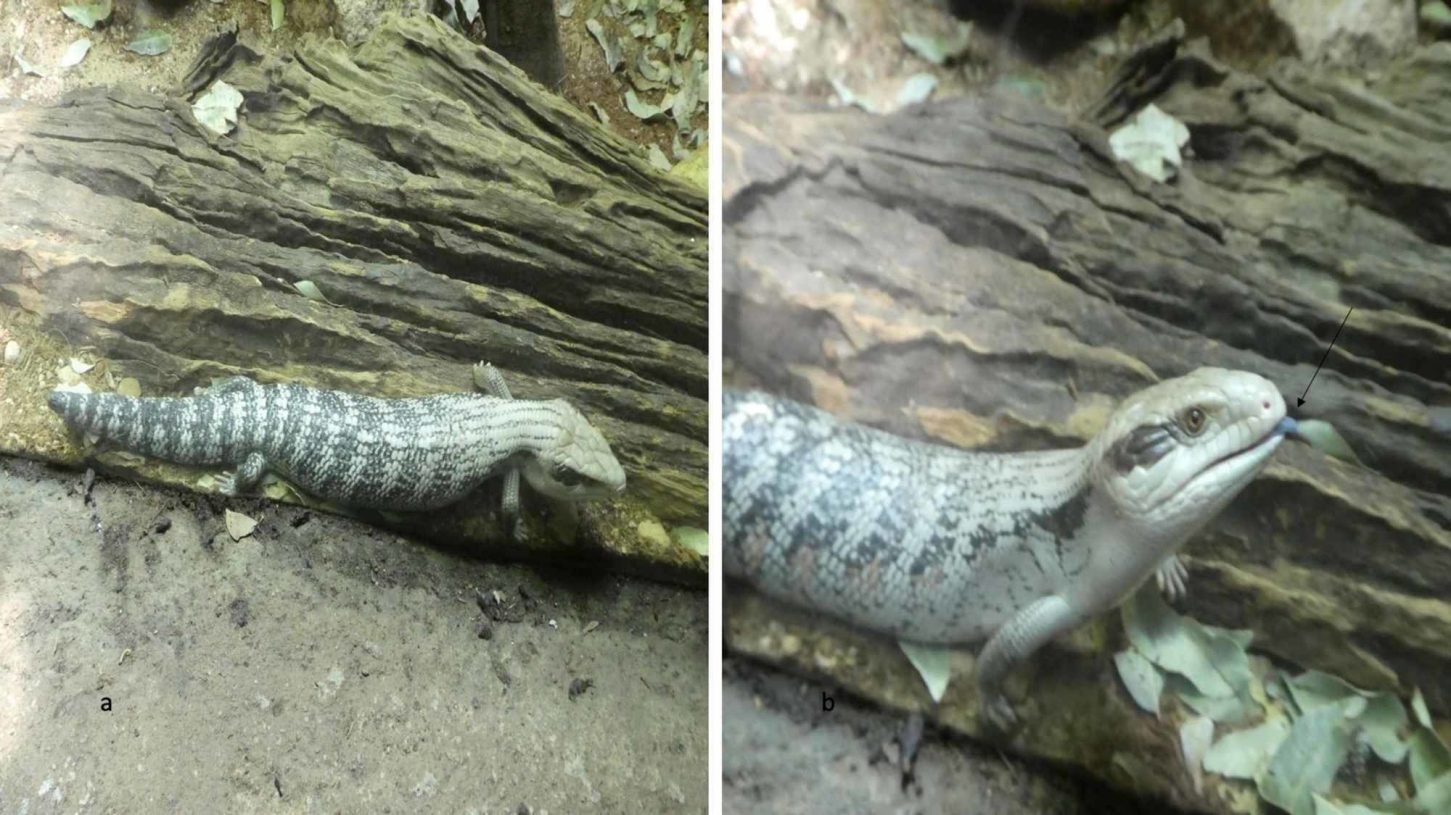
A blue-tongued skink The blue-tongued skink is a lizard that is native to Australia, Indonesia, and New Guinea The skink bares its blue tongue (black arrow) as a warning to potential enemies Source: photographs taken by the author at Taronga Zoo Sydney, Sydney, Australia

Blue tongues are not limited to humans. The tongues of several animals are also blue or blue-black, e.g., blue-tongued skink (Figure 2), chow-chow dog, giraffe, impala, Jersey cattle, okapis, polar bear, and Sha-Pei dog. Some investigators postulate that the skink’s tongue color is not substantially influenced by the amount of melanin in its skin, but that it has chromatic qualities similar to ultraviolet-blue skin patches of other lizard species and therefore the tongue tissue is ultraviolet-reflective [18]. 

Cyanosis of the tongue, with a blue appearance, is a characteristic finding of bluetongue disease; other major features of this condition include facial and lingual swelling, fever, and hypersalivation. Bluetongue disease is a viral condition of ruminant animals-mammals that chew and regurgitate their food more than once as they digest it several times in their multi-chambered stomach. This arthropod-transmitted, non-contagious disease caused by bluetongue virus is most commonly observed in sheep and less frequently in antelopes, buffalo, camels, cattle, deer, elk, and goats [19].

Systemic conditions associated with a blue tongue include argyria, blue rubber bleb nevus syndrome, cyanosis, methemoglobinemia, and primary adrenal insufficiency (Table 2) [1,9-13]. In addition, a purple-bluish tongue was observed in association with the cancer recurrence in 66 of 82 women with epithelial ovarian carcinoma in a study [6]. The observation of a purple-bluish tongue was significantly correlated with an increase in their platelet count [6].

**Table 2:** Systemic conditions associated with a blue tongue BRBNS: blue rubber bleb nevus syndrome; MetHgb: methemoglobinemia; PAI: primary adrenal insufficiency; Ref: references

Condition	Characteristics	Ref
Argyria	The generalized condition results from exposure to a silver substrate or silver salt including either ingestion or topical application to the tongue, nasal mucosa (nose drops), or skin (wound dressings). Discoloration (blue-gray, blue-black or blue-green) of sun-exposed areas is most common; however, the dyschromia can involve covered skin sites, nails, oral mucosa, and sclera.	[9]
BRBNS	A sporadic rare vascular disorder resulting from abnormal blood vessel development in the skin and internal organs, such as the gastrointestinal tract, of the body. The term nevus describes the malformed blood vessels that are commonly present at birth or present during early childhood. The skin lesions can be tender with overlying hyperhidrosis; they appear as dark blue, red, purple-red or black. Lesions can also be present on the tongue. Lesions in the intestines can bleed, resulting in anemia, or obstruct the bowel. Venous malformations and skeletal abnormalities may also be associated.	[1]
Cyanosis	Central cyanosis is due to poor arterial oxygenation; it is associated with clubbing and bluish discoloration of the mucous membranes (and particularly warm areas such as the tongue and lips), skin and nails; it occurs in patients with various cardiac, hematologic, and pulmonary conditions. There is either an increased quantity of reduced hemoglobin or abnormal hemoglobin in the red blood cells. In contrast, peripheral cyanosis is due to low output states associated with excessive oxygen extraction and typically shows bluish discoloration in cool areas such as the cheeks, earlobes, nose, and nail beds. However, rarely, a blue tongue has been observed in patients with peripheral cyanosis, such as a 53-year-old woman with a long-standing severe rheumatic tricuspid regurgitation.	[10]
MetHgb	The condition is either congenital (hemoglobin M disease, which is asymptomatic, or Type 1, which only affects red blood cells and only presents with asymptomatic bluish-tinted of the skin or Type 2--also known as cytochrome b5 reductase deficiency--which is characterized by babies who have developmental problems, failure to thrive and typically die during the first year) or acquired. Most patients with acquired methemoglobinemia do not have the congenital type of the condition; however, individuals with the genetic form of methemoglobinemia have a greater chance to develop the acquired type of the disease. Acquired methemoglobinemia clinically presents with cyanosis (blue coloration of the skin—especially the fingers and mucous membranes such as the lips and tongue) and chocolate-brown colored blood and is caused by exposure to certain medicines, chemicals, or foods; it can result in death if not treated immediately.	[11]
PAI	Adrenal insufficiency, referred to as Addison’s disease, can be primary (and results from destruction or dysfunction of the cortex of the adrenal gland possibly caused by either amyloidosis, autoimmune diseases, hemorrhage, infection, injury, metastatic carcinoma or surgical removal) or secondary (and can result from etiologies--such as abrupt termination of chronic corticosteroid therapy instead of tapering off the medication, benign pituitary tumors, inflammation, or prior pituitary surgery—that cause inadequate production of adrenocorticotropic hormone production by the pituitary gland). The symptoms of adrenal insufficiency are often insidious: amenorrhea and diminished sex drive in women, appetite loss, dysphagia, fainting and lightheadedness, fatigue, gastrointestinal symptoms (including abdominal pain, diarrhea, nausea and vomiting), hypotension, joint or muscle pain, psychiatric symptoms (including behavior changes, decreased motivation, depression, irritability, and mood disturbances), salty food craving and weight loss. Therefore, patients may present in acute adrenal failure (Addisonian crisis) with lethargy, shock, hyperkalemia, and hyponatremia. Cutaneous manifestations of adrenal insufficiency include hair (including alopecia with resulting scanty body hair), nail (including brittle, thin and rarely pigmented), and mucocutaneous (including not only ‘bronze’ darkening typically in sun-exposed areas but also hyperpigmentation in sun-protected locations including skin folds such as the axilla, conjunctival mucosa, genitalia and nipples, scars, and sites of friction and pressure such as the knees, knuckles, palmar creases, and soles) changes. Indeed, intraoral—usually brown—pigmentation (that can involve not only the alveolar, buccal, gingival, lip and palate mucosa but also the tongue) may be the presenting symptom of adrenal insufficiency. Albeit less common, a blue tongue has been observed in patients with adrenal insufficiency: a 26-year-old woman evaluated in Kolkata, West Bengal, India and a 49-year-old woman evaluated in Kansas City, Kansas, US.	[12,13]

Benign melanocytic lesions (melanocytic macules) and vascular tumors (angioleiomyoma, hemangioma, and varicosities) can present as blue patches or nodules on the tongue. Menni et al. have described the case of a 3-day old White boy with three blue-black, smooth, non-blanchable, and non-palpable macules noted on the left side of the dorsal tongue. Biopsy, at one month of age, showed a focal increase of melanin in the basal cells and some melanophages of the lamina propria. The lesions were unchanged at a follow-up examination five months later [2].

Oral angioleiomyoma most commonly occurs on the lip and palate. However, 9% of the tumors occur on the tongue. Brooks et al. have described the case of a 54-year-old Black woman who presented with a 10-mm rubbery bilobed raised tongue tumor of ten years' duration that was whitish on the dorsum with a bluish ventral mucosa. Biopsy showed bundles of smooth muscle cells surrounding numerous vascular spaces, establishing the diagnosis of angioleiomyoma [3].

A hemangioma is a benign developmental anomaly consisting of vascular channels lined by endothelial cells. They are asymptomatic red or blue lesions that typically appear in infancy, grow rapidly and regress spontaneously as the patient gets older. Tongue hemangiomas usually extend deeply between tongue’s muscles and can cause clinical problems such as bleeding, chewing difficulty, recurrent trauma (from tongue biting and toothbrushing), and upper-airway obstruction [4].

Varicosities are more common in older individuals. They present as blue, red, or purple serpiginous elevations on the ventrolateral surface of the tongue. However, it can appear as a bluish-purple nodule if a thrombus is present within varix [5].

Blue reactive lesions of the tongue include intravascular papillary endothelial hyperplasia and mucocele. An intravascular papillary endothelial hyperplasia is a non-neoplastic reactive intravascular proliferation of endothelial cells from the walls of veins; the benign vascular lesion is associated with a thrombus that is undergoing organization and recanalization. Clinically, intravascular papillary endothelial hyperplasia of the tongue present as nodules that are bluish to red in color and covered by normal mucosa [7].

Dental amalgam consists of a mixture of copper, mercury, silver, tin, and zinc. Amalgam tattoos appear blue, blue-gray, gray, or black and result from the traumatic implantation of the metal into the oral soft tissue. Hence, amalgam tattoos have been observed on the buccal mucosa, the gingiva, the palate, and the tongue [14].

Systemic medications can cause a blue tongue; these include minocycline and some of the drugs typically used to treat psychiatry patients [15-17]. Several patterns of minocycline-induced pigmentation have been observed: at sites of prior (facial acne) scars (type I), on normal skin of arms and legs (type II) and on the sun-exposed area (type III). In addition, the nails, sclera, and oral mucosa, including the tongue, can develop blue (or shades of blue, black, brown, or gray) dyschromia [17].

Blue tongues have been observed in individuals treated with haloperidol or dopamine antagonists. The tongue of a patient receiving large doses of haloperidol (a butyrophenone agent) was observed to be blue [16]. In addition, a “strikingly blue” tongue-referred to as the blue-tongue sign-was observed in two additional patients who were being treated with a selective D2 dopamine antagonist [15,16].

Alroe et al. have described the case of a 19-year-old woman with nausea and headache who received intramuscular prochlorperazine (a phenothiazine dopamine-receptor antagonist) along with intramuscular metoclopramide (a selective D2 dopamine antagonist). Her tongue became blue and swollen with partial obstruction of her upper airway. However, her tongue returned to its normal color and size with a resolution of the respiratory distress after she was treated with benztropine [16].

Yun et al. have described the case of a 23-year-old man with schizophrenia who also developed the blue-tongue sign. He initially received risperidone (a selective D2 dopamine antagonist) while hospitalized; amisulpride (a dopamine D2-receptor antagonist and a dopamine D3-receptor antagonist) was subsequently added. His tongue was noticed to be blue at three weeks of discharge; however, its normal color spontaneously returned without the cessation of his antipsychotic medications [15].

Systemic absorption of FD&C blue dye no. 1 has previously been observed to cause blue discoloration of the skin and nails in a critically ill 79-year-old woman who received total parenteral nutrition through a percutaneous gastrojejunal tube. Blue dye had been added to enteral feeding bags prior to starting the infusion in order to determine if there was an aspiration of gastric contents. The blue skin and nails appeared within two-and-a-half weeks after starting the dye. The dye was discontinued, and the blue color disappeared during the next month [20].

The reported patient developed a blue tongue secondary to exposure to the FD&C blue dye no. 1 used in a gelato. The diagnostic clues to the exogenous etiology included not only the focal blue staining of the upper lip but also the sparing the tip and lateral aspects of the tongue. His lingual dyschromia resolved spontaneously.

## Conclusions

Lingual dyschromia can present as a blue tongue. The features of a man who developed asymptomatic and temporary blue dyschromia of his tongue after contact with FD&C blue dye no. 1 are described in this study. The differential diagnosis of blue dyschromia of the tongue includes not only endogenous and exogenous acquired conditions but also congenital etiologies.
